# Amplifying Voices: Workforce-Related Barriers and Facilitators to Person-Centered Care in LTSS

**DOI:** 10.1093/geroni/igaf122.006

**Published:** 2025-12-31

**Authors:** Philip Taylor, Jennifer Morgan, Deke Cateau

## Abstract

Workforce challenges in long-term care highlight the necessity of improving job quality within the sector, where workers’ voices are crucial. The diverse long-term care workforce poses significant questions about how all perspectives and concerns are represented. For many workers, particularly those on atypical contracts with low pay and minimal skill requirements, the opportunity to express their views on issues affecting their working lives is limited. Nevertheless, providing a meaningful voice is essential for cultivating a culture of empowerment, enabling workers to influence matters that are important to them. Amplifying workers’ voices benefits organizations by enhancing workforce engagement, reducing absenteeism, and fostering innovation. From a social justice standpoint and within contemporary understandings of work, having a voice is considered a fundamental individual right that adds meaning to work. Therefore, facilitating this voice is central to promoting fairness and transparency in long-term care organizations, ultimately benefiting society. However, critical knowledge gaps exist regarding long-term care’s current capabilities to enable employee voice amidst often conflicting demands. Drawing from the perspectives of leaders and workers across various long-term care settings and international evidence, the session will contribute new empirical evidence relating to job quality and provide key insights for human resource practice and public policymakers undertaking sector reforms. Reflections from those engaged in the everyday practice of care delivery will test the applicability of the findings.

